# bwimage: A package to describe image patterns in natural structures

**DOI:** 10.12688/f1000research.19801.3

**Published:** 2020-04-14

**Authors:** Carlos Biagolini-Jr., Regina H. Macedo

**Affiliations:** 1 Programa de Pós-Graduação em Ecologia, Universidade de Brasília, Brasília, DF, 70910-900, Brazil; 2 Departamento de Zoologia, Universidade de Brasília, Brasília, DF, 70910-900, Brazil

**Keywords:** animal, ecology methods, field, image analyses, image processing, vegetation patterns

## Abstract

Currently R is the most popular software for data analyses among biologists. Here, we present bwimage, a package designed to describe patterns from black and white images. The package can be used for a wide range of applications. We implemented functions previously described in the literature to calculate parameters designed originally, but not exclusively, for vegetation structures. Additionally, we propose a new parameter: the aggregation index. We demonstrate applications for field work, providing examples that range from calculation of canopy openness, description of patterns in vertical vegetation structure, to patterns in bird nest structure. We provide advice and illustrated examples of how to produce high quality images for analyses.

## Introduction

The facility to obtain high quality digital images creates the opportunity to measure natural variables using image analyses. Black and white pictures have frequently been used to understand patterns in field ecology, especially in plant biology studies ^1^. However, the use of plant image analyses software is not easily extended to other biological fields for several reasons. Free programs are uncommon and paid software normally has threshold algorithms that were specifically designed for vegetation pictures ^2^. Thus, a flexible method that would allow the application of such analyses to other subjects would be welcome. For example, despite the relatively well reported descriptions of bird nests and egg morphology in Del Hoyo and collaborators ^3^ (but see Xiao, Hu ^4^), there are no well-established approaches to estimate nest wall openness patterns.

Currently, R software ^5^ allows users to migrate from data processing based on combinations of different software (with the possibility of having costly licensing, software-specific files, incompatibility between operating systems and lack of updates) to a free, single cross-platform software. Here, we introduce bwimage, a package for R that can be used to analyze patterns in black and white images from natural structures. We provide data examples for applications and descriptions of routines for processing of black and white images.

## Methods

### Implementation

**Figure 1.**
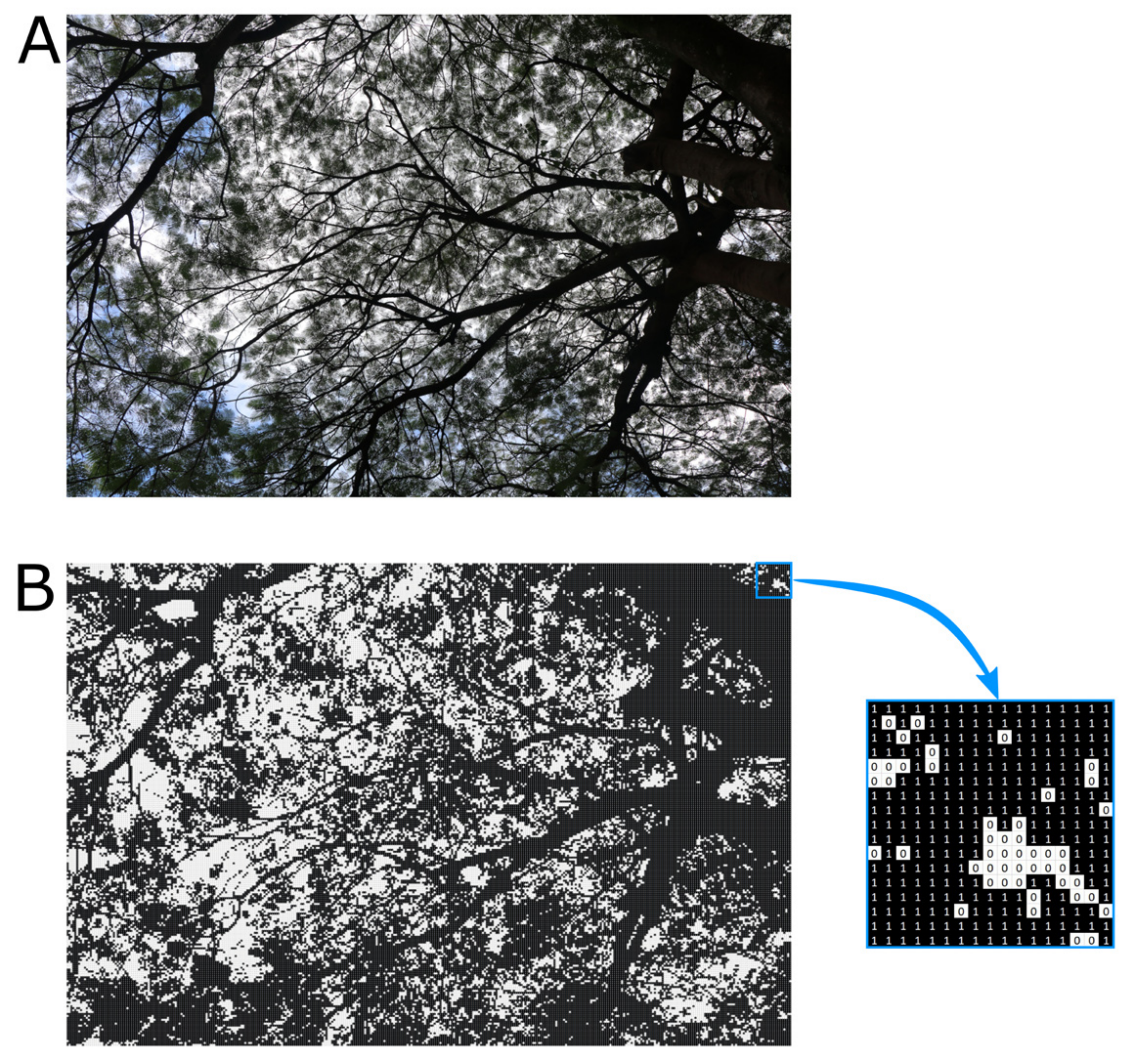
General approach for image analysis in the bwimage package. **A**) An image of a natural structure is obtained with digital photography; here we used an image from a canopy. **B**) The image is converted into a binary matrix, functions threshold_color (to a single image) or threshold_image_list (for two or more images). In the data matrix the value one represents black pixels, zero represents white pixels and NA represents transparent pixels.

**Figure 2.**
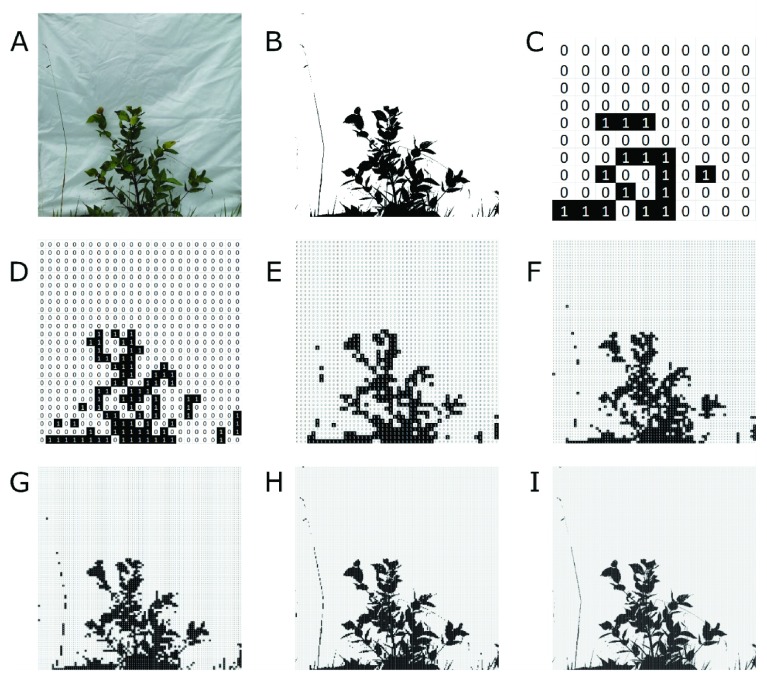
Comparison of different resamplings from a figure of 2500×2500 pixels. **A**) Original image. **B**) Black and white conversion of the original image by GIMP software, i.e. all pixels converted to either black or white in image. **C**– **I**) conversion of the original image to binary matrices of 10×10, 50×50, 75×75, 100×100, 250×250, and 500×500, respectively.

Bwimage´s analysis of images is based on the transformation from a picture (“jpeg” and “png” files are allowed) to a binary matrix ( Figure 1). For each pixel, the intensity of red, green, blue, or the average of these three channels (argument channel) is compared to a threshold (argument threshold_value). If the average intensity is less than the threshold (default is 50%) the pixel will be set as black, otherwise it will be white. Beyond RGB intensity in PNG images, the alpha channel is used to set transparent pixels, i.e *.* alpha channel values above the threshold (argument threshold_value; default is 50%) will set the pixel as transparent. In the data matrix, the value one represents black pixels, zero represents white pixels and NA represents transparent pixels. For high resolution files, i.e. numbers of pixels in width and height, we suggest reducing the resolution to create a smaller matrix, as this strongly reduces GPU usage and time necessary to run analyses. However, by reducing resolution, the accuracy of data description will also be lowered. Figure 2 compares different resamplings from a figure of 2500×2500 pixels. If the user is not acquainted with scale and threshold processing and/or images were captured under different light conditions, we recommend the scale and application of threshold algorithms in a native image editor software, such as GIMP ^6^, and subsequent usage of the resulting images with the bwimage package.

**Figure 3.**
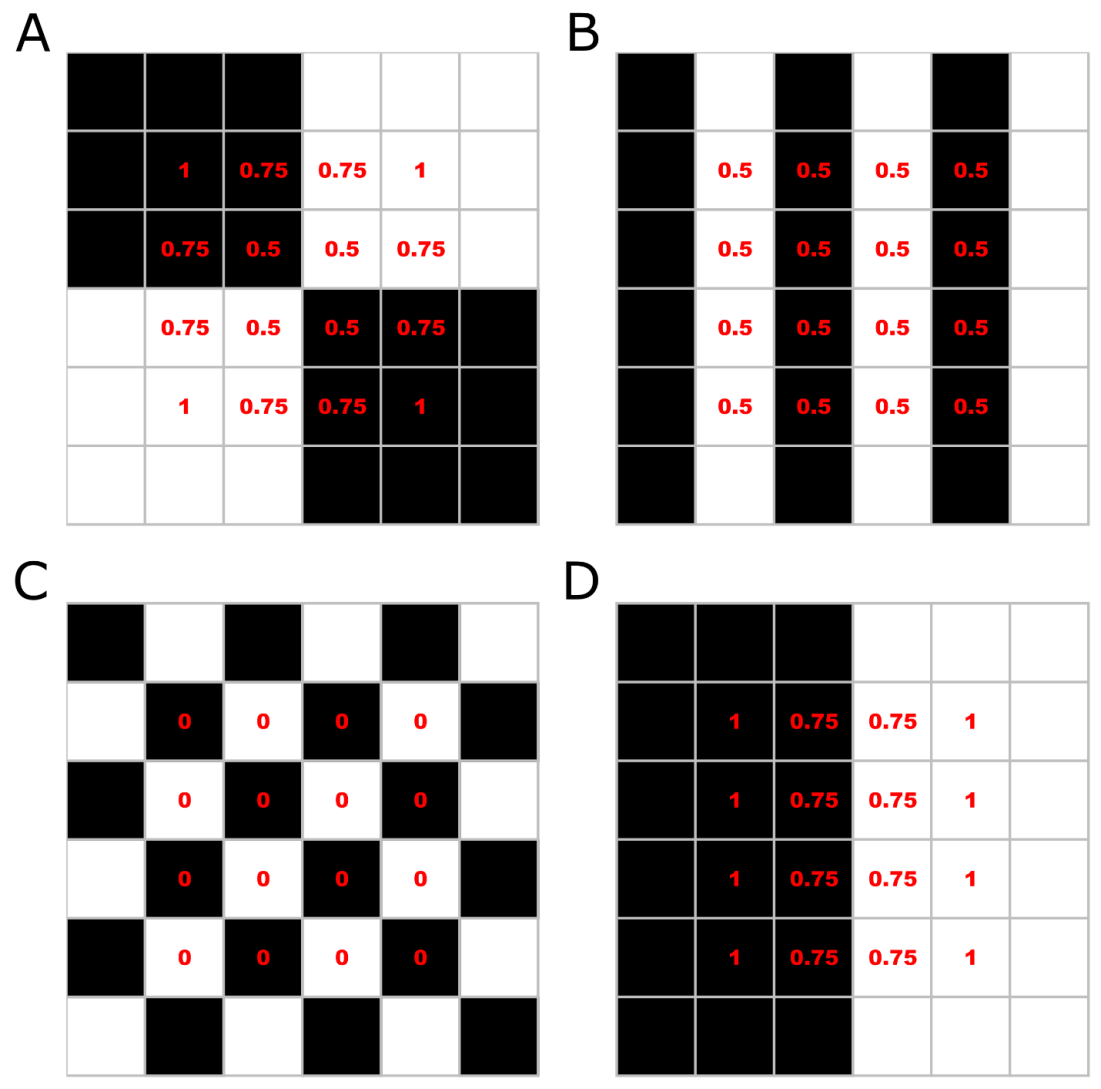
Demonstration of aggregation index calculation. Images represent photos of 6×6 pixel, with 50% black and 50% white pixels. For the aggregation index calculation, first, the proportion of same-color neighboring pixels (SCNP) is calculated (marginal lines and columns are excluded). Then, SCNP are averaged. In these examples, the average SCNP is 0.75 ( **A**), 0.5 ( **B**), 0 ( **C**) and 0.875 ( **D**). Next, the maximum and minimum possible aggregation indexes are calculated. In images with 50% black and white pixels, the minimum aggregation will be pixels distributed in chessboard style ( **C**), and the highest aggregation will be the aggregation of all same color pixels on each image side ( **D**). Thus, by scaling the aggregation by the minimum and maximum possible aggregation, the final aggregation index is: 0.857 ( **A**), 0.571 ( **B**), 0 ( **C**) and 1 ( **D**).

Several metrics can be performed with the functions presented in Table 1. We implemented functions to calculate parameters designed originally, but not exclusively, for vegetation structures (described by Zehm *et al.* 2003) and propose a new parameter: the aggregation index. The aggregation index is a standardized estimation of the average proportion of same-color pixels around each image pixel. First, the proportion of same-color neighboring pixels (SCNP) is calculated (marginal lines and columns are excluded). Next, the SCNP for all pixels are averaged; then, given the proportion of black and white pixels, number of pixels in height and width, and location of transparent pixels (when present), the maximum and minimum possible aggregation indexes are calculated. Finally, the observed aggregation is standardized to a scale where the minimum possible value is set at zero and the maximum value is set at one ( Figure 3).

**Table 1.** Main functions of the bwimage package.

Functions	Description
**Image processes**	
threshold_color	Convert an image into a matrix
threshold_image_list	Convert several images into a list of matrices
image_information	Summary of image information
compress	Map data from square to circular image matrix
stretch	Map data from circular to square image matrix
**Image analyses**	
denseness_total	Proportion of black pixels in relation to all pixels
denseness_row	Proportion of black pixels in rows subsets
denseness_column	Proportion of black pixels in column subsets
hole_row	Description of white pixel continuous sequences in rows
hole_columm	Description of white pixel continuous sequences in columns
hole_section_data	Summary information of holes of a given color in a given section
light_gap	Left and right distances from first black pixel to image edge
heigh_maximum	Higher black pixel in all image
altitudinal_profile	Description of higher black pixel in image sections
heigh_propotion	Height below which a given proportion of vegetation denseness occurs
topline	Line running along the crest of highest black pixel
aggregation_index	Pixel aggregation estimator

### Operation

Bwimage is written in the R programming language ^5^, and can be run on Windows, Mac OS X, and Linux systems. The package is available at the CRAN repository, and the development releases are available at Github (see *Software availability*) ^7^. The bwimage CRAN page documents package dependencies. Input images must be in one of the following formats PNG, JPG, or JPEG.

## Use cases

Canopy openness is one of the most essential ecological parameters for a field ecologist. In the bwimage package, canopy openness can be calculated based on a single picture. To illustrate, we demonstrate below how to analyze a canopy image with the bwimage package. The photo was taken with a digital camera placed in the ground, perpendicular to the ground. Canopy closure can be calculated by estimating the total amount of vegetation in the canopy. Canopy openness is equal to one minus the canopy closure. For this example, we used the original image from Figure 1. The original image file is provided as *Underlying data* ^8^.


canopy_matrix<-
threshold_color("canopy.JPG",compress_method="proportional",compress_rate=0.1)

1-denseness_total(canopy_matrix)
[1] 0.1297333


Several metrics to describe vertical vegetation complexity can be performed by the bwimage package (see Table 1). Here we provide examples based on an image ( Figure 2A) from a vegetation plot of 30×100cm ^1^. The original image file is provided as *Underlying data* ^9^. On the 100cm side of this plot we placed a panel of 100×100 cm, covered with white cloth, and perpendicular to the ground. A plastic canvas of 50x100cm was used to cover the vegetation along a narrow strip in front of a camera positioned on a tripod at a height of 55 cm. A photograph of the portion of standing vegetation against the white cloth was taken.


vegetation_matrix<-threshold_color(bush)

denseness_total(vegetation_matrix,height_size = 100, width_size = 100)
[1] 0.115248

topline(vegetation_matrix)
topline785.6

heigh_propotion_test(vegetation_matrix,proportion=0.75,height_size=100)
Height below which 0.75 of the vegetation denseness is located
31.2aggregation_index(vegetation_matrix)
adjusted_aggregation non_adjusted_aggregation
0.8512586                0.9634373


**Figure 4.**
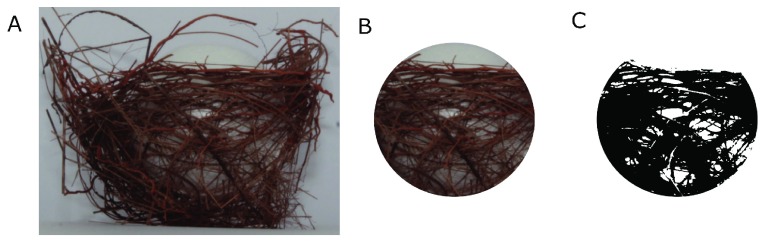
Description of how to produce an image to evaluate patterns in bird nest wall openness. The following example used a blue-black grasssquit ( *Volatinia jacarina*) nest deposited in the museum collection Coleção Ornitológica Marcelo Bagno, at Universidade de Brasília (catalog number COMB-N682). **A**) A photo of the nest was taken with a white styrofoam ball (50mm in diameter) placed inside the nest chamber. **B**) In GIMP software (version 2.10.10), we added alpha channel (to allow transparent pixels), and used the Ellipse select tool to select contrast of the white ball and nest wall, and then erased all background elements. **C**) To ensure that shadows from nest wall did not influence white ball detection, we also performed the threshold processing in GIMP. To preserve the transparent pixels from background elements, the final image must be saved as a PNG file.

Variation in eggs and nest morphology provide relevant information concerning bird life history that has frequently been used to answer ecological ^10– 12^ and evolutionary questions ^13– 15^. Here we analyze examples that address the quantification nest wall openness and the aggregation of nest wall holes, using a nest of the blue-black grasssquit ( *Volatinia jacarina*) deposited in the museum collection Coleção Ornitológica Marcelo Bagno, at Universidade de Brasília (register number COMB-N682). Figure 4 describes how to produce a high-quality image to describe patterns in bird nest wall openness. The original image file used is provided as Underlying data ^16^.


NestWall_matrix<-threshold_color("NestWall.png",filetype="png",compress_method="width_fixed",target_width=300)

denseness_total(NestWall_matrix)
[1] 0.7612406

aggregation_index(NestWall_matrix)
adjusted_aggregation non_adjusted_aggregation
0.9007821                0.9502800


## Conclusions

The bwimage package provides accessible and simple methods for ecologists and field researchers to describe patterns from black and white digital images. It is a flexible method that allows the application of image analyses to an exceptionally broad range of research subjects. Bwimage´s analysis is based on a simple computational routine based on the transformation of a picture (“jpeg” or “png” files) into a binary matrix, followed by the analysis itself. Several metrics can be calculated by the bwimage package. We implemented functions previously described in the literature, and additionally, we proposed a new parameter: the aggregation index, which generates a standardized estimate of the average proportion of same-color pixels around each image pixel. The application of this methods is exceptionally broad.

## Data availability

### Underlying data

Figshare: Canopy of Royal poinciana. https://doi.org/10.6084/m9.figshare.8429117.v2 ^8^

Figshare: Image from a vegetation plot of 30x100cm. https://doi.org/10.6084/m9.figshare.8429882.v2 ^9^

Figshare: Blue-black grassquit (Volatinia jacarina) nest. https://doi.org/10.6084/m9.figshare.8432018.v1 ^16^

Data are available under the terms of the Creative Commons Zero "No rights reserved" data waiver (CC0 1.0 Public domain dedication).

## Software availability

- Software available from: https://CRAN.R-project.org/package=bwimage
- Source code available from: https://github.com/biagolini/bwimage
- Archived source code at time of publication: https://doi.org/10.5281/zenodo.3266299 ^7^
- License: GPL-3
