# Supplementary material for: bwimage: A package to describe image patterns in natural structures
Source: F1000Res. 2020 Apr 14;8:1168. Originally published 2019 Jul 23. [Version 3] doi: 10.12688/f1000research.19801.3 (PMC7020331; doi:10.12688/f1000research.19801.3)
Supplement: Peer review history

# Peer review: Reviewer response for version 2

**DOI:** 10.5256/f1000research.23161.r59116

**Published:** 2020-02-13

**Authors:** Roy Francis

**Affiliations:** 1 Department of Medical Biochemistry and Microbiology, Uppsala University, Uppsala, Sweden; 2 National Bioinformatics Infrastructure Sweden, Uppsala, Sweden

- Describing image patterns in natural structure is not my area, therefore my comments are quite general.
- I have tried running the tool on two different OS and tried out most of the functions using several images. Generally, the tool and functions work as described. The documentation is reasonably well written and easy to follow.
- R is quite lacking in image analysis, therefore this package could be a useful little addition.
- For effectively following the workflow described here, optimal thresholding is the key. The thresholding offered with the package is too basic and the user has to rely on external tools.
- To compare the resulting metrics, two images need to be exactly comparable. The zoom/crop/scaling of the image, the lighting conditions, shadow etc would most likely render the results incomparable. This is something to be handled by external tools as well.
- My opinion is that image recognition and classification has to be implemented as some sort of machine learning algorithm rather than any manual thresholding for practical real world application. See relevant example ^1^.
- No implementation to work with large images (Sparse matrices etc). Only available option is to scale down the image and work with a smaller image. Issues with small image size is briefly discussed in the manuscript.
- Summary functions would be nice. Some simple functions to visualise the results would be nice. For example just to preview the thresholded image, I used image(t(apply(img_thres,2,rev))).
- Benchmarking: It's hard to say how good the results are. It would've been nice to see a comparison of this tool to other existing tools in terms of efficiency as well as accuracy. For example; How does SCNP compare to other measures of aggregation (used in this field)? It would be nice to see some sort of use case on real world dataset rather than a few isolated images.
- A detailed HTML vignette showcasing all the functionality and typical use case workflows. The r-bloggers tutorial was a good start but I wouldn't consider that as a stable location. Perhaps on github along with the source?
- Possible typos in function names: heigh_maximum, heigh_propotion, heigh_propotion_test, hole_columm. At least, it's consistent with Table 1 in the manuscript.
- In figure 2, dimension of sub plot I is missing in the caption.
- Figure 3 caption typo: Imagens.
- Typo Page 8, last line of acknowledgements. acknowledg.

Are the conclusions about the tool and its performance adequately supported by the findings presented in the article?

Yes

Is the rationale for developing the new software tool clearly explained?

Yes

Is the description of the software tool technically sound?

Yes

Are sufficient details of the code, methods and analysis (if applicable) provided to allow replication of the software development and its use by others?

Yes

Is sufficient information provided to allow interpretation of the expected output datasets and any results generated using the tool?

Partly

Reviewer Expertise:

Molecular Biology, Bioinformatics

I confirm that I have read this submission and believe that I have an appropriate level of expertise to confirm that it is of an acceptable scientific standard.

## References

[1] : Automatic detection of woody vegetation in repeat landscape photographs using a convolutional neural network. *Ecological Informatics*.2019;50: 10.1016/j.ecoinf.2019.01.012 220-233 10.1016/j.ecoinf.2019.01.012

# Peer review: Reviewer response for version 2

**DOI:** 10.5256/f1000research.23161.r55892

**Published:** 2019-11-12

**Authors:** Francesco Chianucci

**Affiliations:** 1 The Council for Agricultural Research and Economics (CREA) - Research Centre for Forestry and Wood, Arezzo, Italy

I think the author(s) has made the required correction and therefore I suggest the revised article is ready for indexing.

Are the conclusions about the tool and its performance adequately supported by the findings presented in the article?

Partly

Is the rationale for developing the new software tool clearly explained?

Yes

Is the description of the software tool technically sound?

Yes

Are sufficient details of the code, methods and analysis (if applicable) provided to allow replication of the software development and its use by others?

Partly

Is sufficient information provided to allow interpretation of the expected output datasets and any results generated using the tool?

Partly

Reviewer Expertise:

Forestry

I confirm that I have read this submission and believe that I have an appropriate level of expertise to confirm that it is of an acceptable scientific standard.

# Peer review: Reviewer response for version 1

**DOI:** 10.5256/f1000research.21722.r51562

**Published:** 2019-08-13

**Authors:** Francesco Chianucci

**Affiliations:** 1 The Council for Agricultural Research and Economics (CREA) - Research Centre for Forestry and Wood, Arezzo, Italy

The topic is interesting. However, I think the package makes simple things which many other similar packages do, and omit some intermediate steps required to refine the analysis.

 First, the authors should provide a list of thresholding (either single or dual) methods to binarize images. The single Otsu, the minimum algorithm, the two-corner method are some of the algorithm which should be developed. The package could also use some dependancies from other packages to implement these thresholding. For instance, the rtiff package contains the function autoThreshold which implement the Ridler-Calvard thresholding. I also suggest take a look at the caiman package which contains tool for canopy image analysis, see also below.

 Second, users can also process single channel image or decide to use a single channel (for example, the blue channel is frequently used for canopy images). So the user should have flexibility on choosing the image feature to process (setting the channel, setting the threshold).

 Canopy images are usually fish eye images, and therefore they require correction for lens projection. In addition, canopy openness should be weighted for zenith angle in fish eye image. Similarly, gap fraction is required for zenith ring. Thus, the packages should allow to work with fish eye (circular image), namely correct for lens distortion, setting the circular inner mask, extract information for zenith angle ranges (inner rings). I suggest to take a look in the caiman package to inherit its functions.

 An interesting attribute from the packages is the identification of row or column gaps (holes), namely continuous sequences of white pixels. In my view, a very interesting output would be the identification and the labelling of all holes in the image, along with their summary statistics (e.g. number of holes, size of each hole in number of pixels, average, sd and so on). I think the package will improve strongly if authors can implement such function. This would allow the extraction of canopy attributes from images ^1^.

Are the conclusions about the tool and its performance adequately supported by the findings presented in the article?

Partly

Is the rationale for developing the new software tool clearly explained?

Yes

Is the description of the software tool technically sound?

Yes

Are sufficient details of the code, methods and analysis (if applicable) provided to allow replication of the software development and its use by others?

Partly

Is sufficient information provided to allow interpretation of the expected output datasets and any results generated using the tool?

Partly

Reviewer Expertise:

Forestry

I confirm that I have read this submission and believe that I have an appropriate level of expertise to confirm that it is of an acceptable scientific standard, however I have significant reservations, as outlined above.

## References

[1] : An objective image analysis method for estimation of canopy attributes from digital cover photography. *Trees*.2018;32(3) : 10.1007/s00468-018-1666-3 713-723 10.1007/s00468-018-1666-3

# Response

**Published:** 2019-10-16

**Authors:** Carlos Biagolini-Jr.

Dear Dr. Francesco Chianucci,

We appreciate all of your helpful comments, and we are certain that they have improved significantly the original version of the Package. In the new version (1.2) of bwimage package published in CRAN we: i) introduced two new functions (stretch and compress functions) to provide an additional tool to distort images. These functions are applications of algorithms for mapping images from circle to square, and vice versa, adapted from Lambers (2016 *J of Computer Graphics Techniques*); and ii) included the possibility of users processing single channel images.

We considered incorporating other packages for threshold in bwimage dependencies; however, we decided to avoid a cross-dependency of packages because it implies in a heavy load package, and brings possible compatibility issues with R updates.  Thus, we chose to create a tutorial (shared on https://www.r-bloggers.com/using-bwimge-r-package-to-describe-patterns-in-images-of-natural-structures/) to provide an overview of bwimage package and demonstrate examples of how to apply threshold algorithms from the package autothresholdr, following its application in bwimage package. A comparison of estimation of vegetation density from a bush image submitted to different thresholding algorithms is provided. Note: the current version of autothresholdr package (1.3.5) provide 17 threshold algorithms by function auto_thresh, covering a wide range of applications.

We do not mention in the previous version of this article, but bwimage version 1.0 already had a function (hole_section_data) to summarize holes statistics (i.e. number of holes, mean hole size, sd, minimum and maximum size).  This function was designed to be used inside a loop or apply-family functions. By combination of hole_section_data and loop function, users can potentially collect a summary of holes statistics. The size of each section is obtained by the hole_section function, which returns size and map of each hole. We add information about this function in manuscript table 1. We also provide an example of how to apply hole_section_data for a set of 12 images in the above-mentioned tutorial.

With my best regards.

Sincerely,

Carlos Biagolini-Jr
